# The Multi-Targeted Kinase Inhibitor Sunitinib Induces Apoptosis in Colon Cancer Cells via PUMA

**DOI:** 10.1371/journal.pone.0043158

**Published:** 2012-08-17

**Authors:** Jing Sun, Quanhong Sun, Matthew F. Brown, Crissy Dudgeon, Julie Chandler, Xiang Xu, Yongqian Shu, Lin Zhang, Jian Yu

**Affiliations:** 1 University of Pittsburgh Cancer Institute, Departments of Pathology, and Pharmacology and Chemical Biology, University of Pittsburgh School of Medicine, Pittsburgh, Pennsylvania, United States of America; 2 Department of Medical Oncology, The First Affiliated Hospital of Nanjing Medical University, Nanjing, Jiangsu, People’s Republic of China; 3 Research Institute of Surgery and Daping Hospital, The Third Military Medical University, Chongqing, People’s Republic of China; H.Lee Moffitt Cancer Center & Research Institute, United States of America

## Abstract

Constitutive activation of pro-survival kinases has become a promising target of small molecules with an increasing interest in developing multi-targeted agents. The mechanisms underlying the responsiveness to most agents targeting cancer specific survival pathways are still poorly understood but critical for their clinical application. In this study, we found that sunitinib, a small molecule inhibitor of multiple tyrosine kinases including VEGFRs and PDGFRs induces apoptosis and inhibits cell growth in colon cancer cells in cell culture and xenograft models via the BH3-only protein PUMA. Sunitinib treatment induced *PUMA* transcription via the AKT/FoxO3a axis. PUMA, BH3 mimetics, or 5-Flurourical sensitized colon cancer cells to sunitinib-induced apoptosis. Furthermore, PUMA was induced by sunitinib treatment in xenograft tumors, and deficiency in *PUMA* significantly suppressed the anti-tumor effects of sunitinib. Our study suggests that PUMA-mediated apoptosis is important for the therapeutic responses to sunitinib, and activation of the mitochondrial pathway by BH3 mimetics or PUMA manipulation may be useful for improving the antitumor activity of sunitinib. Modulation of PUMA and selective Bcl-2 family members might be potential biomarkers for predicting sunitinib responses.

## Introduction

Colorectal cancer (CRC) is the third leading cause of cancer-related death in the US and the incidence is on the rise in developing countries [1]. Even with the combination of improved chemotherapy and radiation in past decades, the 5 year survival of CRC patients with advanced disease remains unacceptably low. Aberrant activation of various kinase pathways is common in most solid tumors, which can lead to increased proliferation, survival, angiogenesis or invasion [2], [3]. In recent years, considerable hope has been placed on agents developed to target oncogenic kinases, whose use in combination with chemotherapy or radiation might improve the survival and outcome of CRC patients [4]. The targeted approach is expected to ultimately deliver safer and more effective cancer therapeutics [5]. One major challenge in the clinical use of these agents is the prevalence of intrinsic and acquired resistance, whose underlying mechanisms remain largely unknown and a subject of intense investigation [4], [5].

Sunitinib (also known as SU11248) was developed as a multi-targeted receptor tyrosine kinase (RTK) inhibitor, and approved by the FDA in 2006 for the treatment of renal cell carcinoma (RCC) and imatinib resistant gastrointestinal stromal tumor (GIST) [6], [7]. Ongoing clinical trials are being conducted to evaluate its efficacy in other tumor types including metastatic colon cancer [7], [8] (http://clinicaltrials.gov/). Sunitinib inhibits a variety of receptor tyrosine kinases (RTKs) that are either mutated or activated in cancer. These include receptors for platelet-derived growth factor (PDGF-R α and β) and vascular endothelial growth factor receptors (VEGFR1, 2 and 3), as well as KIT (CD117), RET, CSF-1R, and flt3 [6], [7]. Sunitinib has been recommended as a second-line therapy in GISTs that developed resistance to imatinib due to secondary mutations in *c-KIT*. Inhibition of angiogenesis, immune modulation and induction of apoptosis has been suggested to mediate the anti-tumor effects of sunitinib [7]. The mechanisms underlying the cell autonomous effect of sunitinib such as cell killing is not well-understood.

Mitochondria-mediated apoptosis plays an important role in the antitumor activities of a wide variety of conventional chemotherapeutic agents as well as targeted therapies [9], [10]. The Bcl-2 family of proteins are the central regulators of mitochondria-mediated apoptosis, which is engaged by the selective activation or induction of the proximal BH3-only members in response to distinct as well as overlapping signals [11], [12]. The BH3-only protein PUMA plays an essential role in p53-dependent and -independent apoptosis in human cancer cells and mice [13], and activates the mitochondrial pathway via the Bcl-2 family member Bax/Bak following neutralizing all members of antiapoptotic Bcl-2 like molecules [14], [15], [16], [17]. DNA damage induced by gamma-irradiation or commonly used chemotherapeutic agents such as 5-fluorouracil (5-FU), adriamycin and etoposide, induce p53-dependent induction of PUMA and apoptosis [15], [18]. Nongenotoxic stresses such as growth factor deprivation, endoplasmic reticulum poisons and a number of kinase inhibitors induce PUMA through a number of other transcription factors including p73, NF-κB and FoxO3a [13], [19], [20], [21], [22], [23].

In the current study, we demonstrated that sunitinib induces PUMA expression independent of p53 in colon cancer cells. The induction of PUMA was mediated by the transcription factor FoxO3a upon inhibition of AKT. *PUMA* deficiency led to resistance to sunitinib-induced apoptosis in cells as well as in xenografts. Our study provides a molecular mechanism of apoptosis induced by this non-selective kinase inhibitor in colon cancer cells, and has important implications for biomarker discovery and potential strategies to overcome resistance.

## Materials and Methods

### Cell Culture and Drug Treatment

Colon cancer cell lines were obtained from ATCC. All cell lines were maintained at 37°C in 5% CO_2_ and cultured in Mycoy’s 5A medium (Invitrogen, Carlsbad, CA) supplemented with 10% FBS (HyClone, Logan, UT), 100 units/ml penicillin and 100 µg/ml streptomycin (Invitrogen, Carlsbad, CA). The somatic knockout cells lines HCT 116 *p53* KO [24], HCT 116 *PUMA* KO [15], DLD1 *PUMA* KO [18], HCT 116 *FoxO3a* stable knockdown (KD) cells and small interfering RNA (siRNA) [19] have been previously described. Anticancer agents or chemicals used in the study include Sunitinib Malate (Cayman Chemical, Ann Arbor, MI), 5-fluorouracil (5-FU), Gossypol (Sigma, St. Louis, MO), HA14-1 (Axxora LLC, San Diego, CA), ABT-737 (Selleck Chemicals LLC, Houston, TX). Stock solutions of all compounds were prepared in DMSO and diluted by culture medium to working concentrations before use. Cells were infected with adenovirus expressing PUMA, Ad-PUMA [15] (20 MOI) alone or with the addition of sunitinib. Transfection of expression constructs of Flag-Mcl-1 [16], Bcl-2 and constitutive AKT (Millipore) was performed as described [20].

### Western Blotting and Subcellular Fractionation

Antibodies used for Western blotting included those against caspase-3, Myc (9B11), FoxO3a (total), p-FoxO3a, AKT (total), p-AKT (S473) (Cell Signaling Technology, Beverly, MA), cytochrome c, α-tubulin, Bcl-xL, Mcl-1 (BD Biosciences), caspase-9 (Stressgen Bioreagents, Ann Arbor, MI), cytochrome oxidase subunit IV (Cox IV, Invitrogen), Bcl-2 (Dako, Carpinteria, CA, USA), Flag (Sigma), PUMA [15], p53, p21, Bim, Bid, Noxa, Smac and β-actin (EMD Biosciences, Gibbstown, NJ). Western blotting was performed as previously described [25].

The release of cytochorme c and Smac was detected in the cytosol following subcelluar fractionation as described [20], [26]. In brief, cells were treated in T75 flasks for indicated times and subject to differential centrifugation to obtain cytoplasmic and mitochondrial fractions. Concentrations of cytosolic fractions obtained were normalized using a protein assay dye reagent (Bio-Rad, Hercules, CA). The fractions were mixed with equal volumes of 2x Laemmli sample buffer and subjected to Western blotting analysis.

### Chromatin Immunoprecipitation

Chromatin immunoprecipitation (ChIP) was done using the Chromatin Immunoprecipitation Assay kit (Millipore, Billerica, MA) with FoxO3a antibody for chromatin precipitation as described [18]. The precipitates were analyzed by PCR using primers 5-GCGCACAGGTGCCTCGGC-3 and 5-TGGGTGTGGCCGCCCCT-3 as described [22].

### Luciferase Assays

Cells were transfected with PUMA reporters containing either WT or mutant FoxO3a binding sites [19], with the transfection control β-galactosidase reporter pCMVβ (Promega), and treated with 12 µM sunitinib for 24 hours. Cell lysates were collected and luciferase activities were measured as previously described [23]. All reporter experiments were done in triplicate and repeated three times.

**Figure 1:**
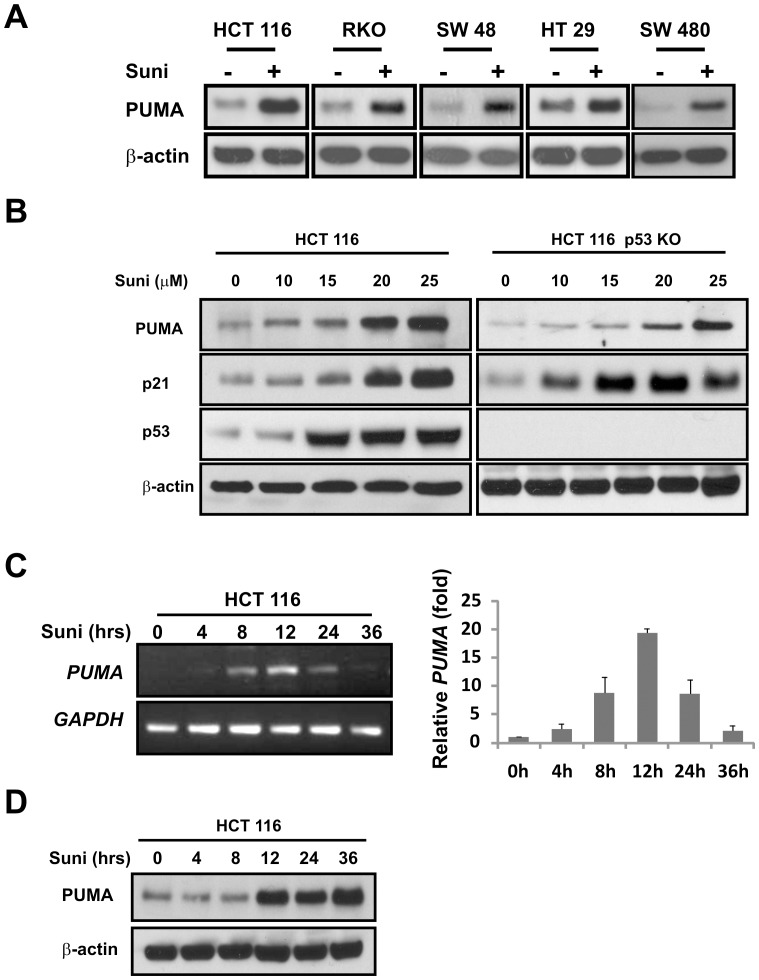
PUMA is induced by sunitinib in colon cancer cells irrespective of *p53* status. (**A**) The indicated colon cancer cell lines were treated with 15 µM sunitinib for 24 hours. The levels of PUMA were analyzed by Western blotting. (**B**) The parental HCT 116 (WT) or *p53* KO cells were treated as in (A) with increasing doses of sunitinib and analyzed for PUMA, p53 and p21 expression. (**C**) **and** (**D**) HCT 116 cells were treated with 15 µM sunitinib for the indicated times. *PUM*A mRNA and protein levels were analyzed by real-time RT-PCR and Western blotting, respectively. *GAPDH* mRNA was used as a control for normalization in RT-PCR, and β-actin was used as a control for loading in Western blotting.

### Real-time Reverse Transcription-PCR

Total RNA was isolated from cells using the Mini RNA Isolation II kit (Zymo Research, Irvine, CA) according to the manufacturer’s protocol. Total RNA (1 µg) was used to generate cDNA using SuperScript II reverse transcriptase (Invitrogen). Real-time PCR was carried out for PUMA and GAPDH as described [22].

**Figure 2:**
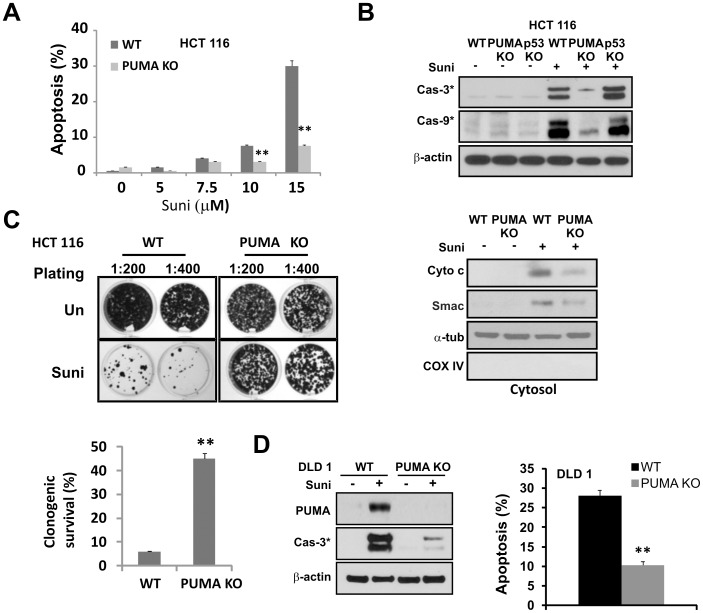
PUMA mediates sunitinib-induced apoptosis in colon cancer cells. (**A**) HCT 116 WT and *PUMA* KO cells were treated with increasing doses of sunitinib for 48 hours. Apoptosis was analyzed by nuclear fragmentation assay. Data were obtained from 3 independent experiments. **, *P*<0.01, KO *vs.* WT. (**B**) HCT 116 cells with the indicated genotypes were treatment with 15 µM sunitinib for 48 hours. *Upper*, the cleavage products of caspase-3 and -9 were analyzed by Western blotting. *Lower*, the release of cytochrome c and Smac in the cytosol was analyzed by Western blotting. β-actin and CoxIV are controls for loading, and cytosolic and mitochondrial fractionations, respectively. (**C**) Colony formation in HCT 116 WT and *PUMA* KO cells treated with sunitinib. Cells were treated by 12 µM sunitinib treatment for 48 hours, then plated at 1∶200 or 1∶400 dilution (∼600 or 300 cells per well) in 12-well plates and allowed to form colonies for 14 days. *Upper,* representative pictures of the colonies. *Lower*, the colonies containing >50 cells were enumerated and relative survival was calculated with untreated cells set at 100%. Data were obtained from 3 independent experiments. **, *P*<0.005, KO *vs.* WT. (**D**) DLD1 WT and *PUMA* KO cells were treatment with 30 µM sunitinib for 48 hours. *Left*, PUMA and the cleavage products of caspase-3 were analyzed by Western blotting. *Right*, apoptosis was analyzed by nuclear fragmentation assay. **, *P*<0.01, KO *vs.* WT.

### Apoptosis Assays

Adherent and floating cells were harvested, stained with Hoechst 33258 (Invitrogen), and analyzed for apoptosis by nuclear staining assay. A minimum of 300 cells were analyzed for each treatment [25], [27]. For colony formation assays, equal number of cells were subjected to various treatments and plated into 12-well plates at different dilutions. Colonies were visualized by crystal violet staining 11 to 14 days after plating as previously described [25], [28]. Each experiment was performed in triplicate and repeated at least twice.

**Figure 3:**
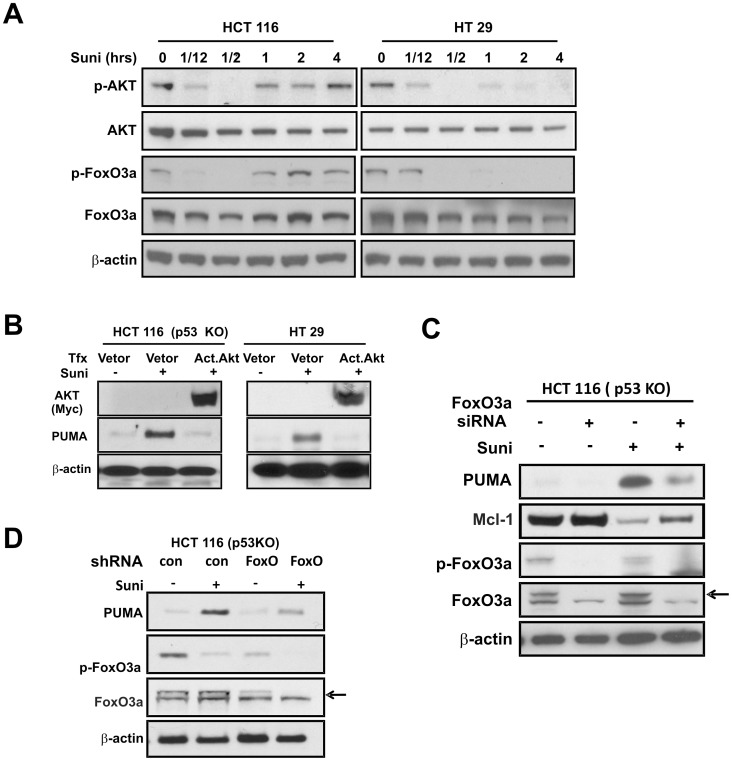
The AKT/FoxO3a axis regulates PUMA induction by sunitinib. (**A**) HCT 116 and HT 29 cells were treated with 15 µM sunitinib for indicated times. The levels of total FoxO3a, phosphorylated(p)- FoxO3a, total-AKT and phosphorylated(p) -AKT (S473) were analyzed by Western blotting. (**B**) HCT 116 *p53* KO cells and HT 29 cells were transfected with either empty vector or a constitutively-active AKT expression constructs for 16 hours, then treated with 15 µM sunitinib for 24 hours. The levels of p-AKT and PUMA were analyzed by Western blotting. (**C**) HCT 116 *p53* KO cells were transfected with either a scrambled siRNA or a *FoxO3a* specific siRNA for 24 hours, and then treated with 15 µM sunitinib for 24 hours. The levels of indicated proteins were analyzed by Western blotting. (**D**) *FoxO3a* stable knockdown (KD) cells were treated with 15 µM sunitinib for 24 hours. The levels of indicated proteins were analyzed by Western blotting. The specific band of total-FoxO3a is indicated by an arrow. β-actin was used as a control for loading.

### Xenograft Tumors

All animal experiments were approved by the Institutional Animal Care and Use Committee (IACUC) at the University of Pittsburgh. HCT 116 WT and *PUMA* KO xenografts were established and measured as described [25]. In brief, 5–6 week old female athymic nude mice (Harlan, Indianapolis, IN) were inoculated with 5×10^6^ cells per site on both flanks. Tumors were allowed to establish for 7 days. The mice were oral gavaged for 10 consecutive days with 80 mg/kg/day sunitinib diluted in sodium citrate buffer (pH 4.7, vehicle), or vehicle [29]. The tumor volumes were measured in two dimensions using a vernier caliper. Mice were randomized into groups, such that the average tumor volume across the groups was the same prior to treatment. For all *in vivo* experiments, tumor volumes were measured every other day in 2 dimensions and volumes were determined in mm^3^ using the formula l×b^2^×0.52 (where l is the larger diameter and b is the smaller diameter of the tumor). Mice were injected i.p. 2 h before sacrifice with a single dose of bromodeoxyuridine (BrdU) at 150 mg/kg to label cells in S phase. BrdU was dissolved in PBS to a final concentration of 30 mg/mL. Histologic and immunofluorescence analysis for apoptosis and proliferation were performed on 5-µM frozen sections, as described [25]. Mice were euthanized 21 days after the treatment, or 24 hours after the third treatment (day 4). Tumors were dissected and snap frozen for western blotting or fixed in 10% formalin before paraffin embedding.

**Figure 4:**
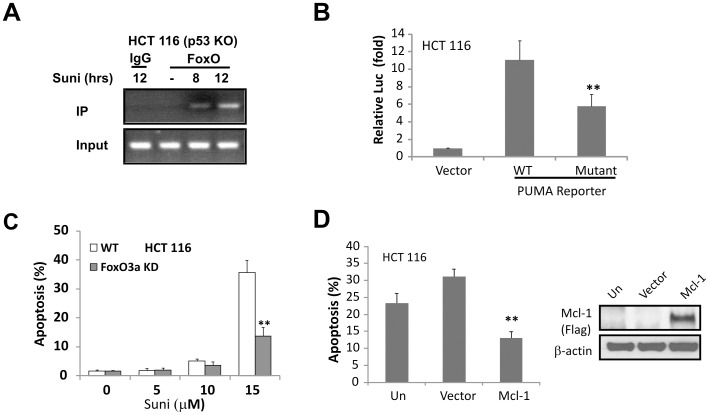
FoxO3a mediates transcriptional activation of PUMA by sunitinib. (**A**) Chromatin immunoprecipitation (ChIP) was performed on HCT 116 *p53* KO cells following 15 µM sunitinib treatment for 8 and 12 hours. IgG was used to as a control for the FoxO3a-specific antibody. (**B**) HCT 116 cells were transfected with the PUMA reporter containing the WT or mutant FoxO3a binding sites for 16 hours, and then treated with 15 µM sunitinib for 24 hours. The reporter activities were measured by luciferase assay as described in methods. (**C**) *FoxO3a* stable knockdown (KD) cells were treated with increasing doses of sunitinib for 48 hours. Apoptosis was analyzed by nuclear fragmentation assay. Data were obtained from 3 independent experiments. **, *P*<0.01, KD *vs.* WT. (**D**) Expression of Mcl-1 suppressed sunitinib-induced apoptosis in HCT 116 cells. Cells were transfected with Flag-tagged Mcl-1 for 16 hours then treated with 15 µM sunitinib for 48 hours. Apoptosis was analyzed by nuclear fragmentation assay. Data were obtained from 3 independent experiments. **, *P*<0.01, KO *vs.* WT. *Right*, Mcl-1 expression was confirmed by Western blotting. β-actin was used as a control for loading.

### TUNEL and Immunostaining

Histological analysis was performed by hematoxylin and eosin staining. Terminal deoxyribonucleotidyl transferase -mediated dUTP nick end labeling (TUNEL) staining on frozen sections was done with recombinant terminal transferase (Roche) and uUTP-Alexa 594 (Invitrogen) according to the manufactures’ instructions, and counterstained with 4′,6-diamidino-2-phenylindole (DAPI) with minor modification made for paraffin samples [25], [30], [31]. Apoptotic cells were counted under a fluorescence microscope in randomly chosen fields, and the apoptosis index was calculated as a percentage of TUNEL-positive cells in at least 1,000 scored cells. BrdU incorporation was visualized with anti-BrdU- Alexa 594 antibody (Invitrogen) and nuclei were visualized with DAPI. The proliferation index was calculated by counting cells under fluorescence microscope in several randomly chosen fields. The BrdU index was calculated as a percentage of BrdU-positive cells in at least 1,000 scored cells. Phospho-AKT, phospho-FoxO3a and active Caspase-3 immunohistochemistry was performed as described [19].

**Figure 5:**
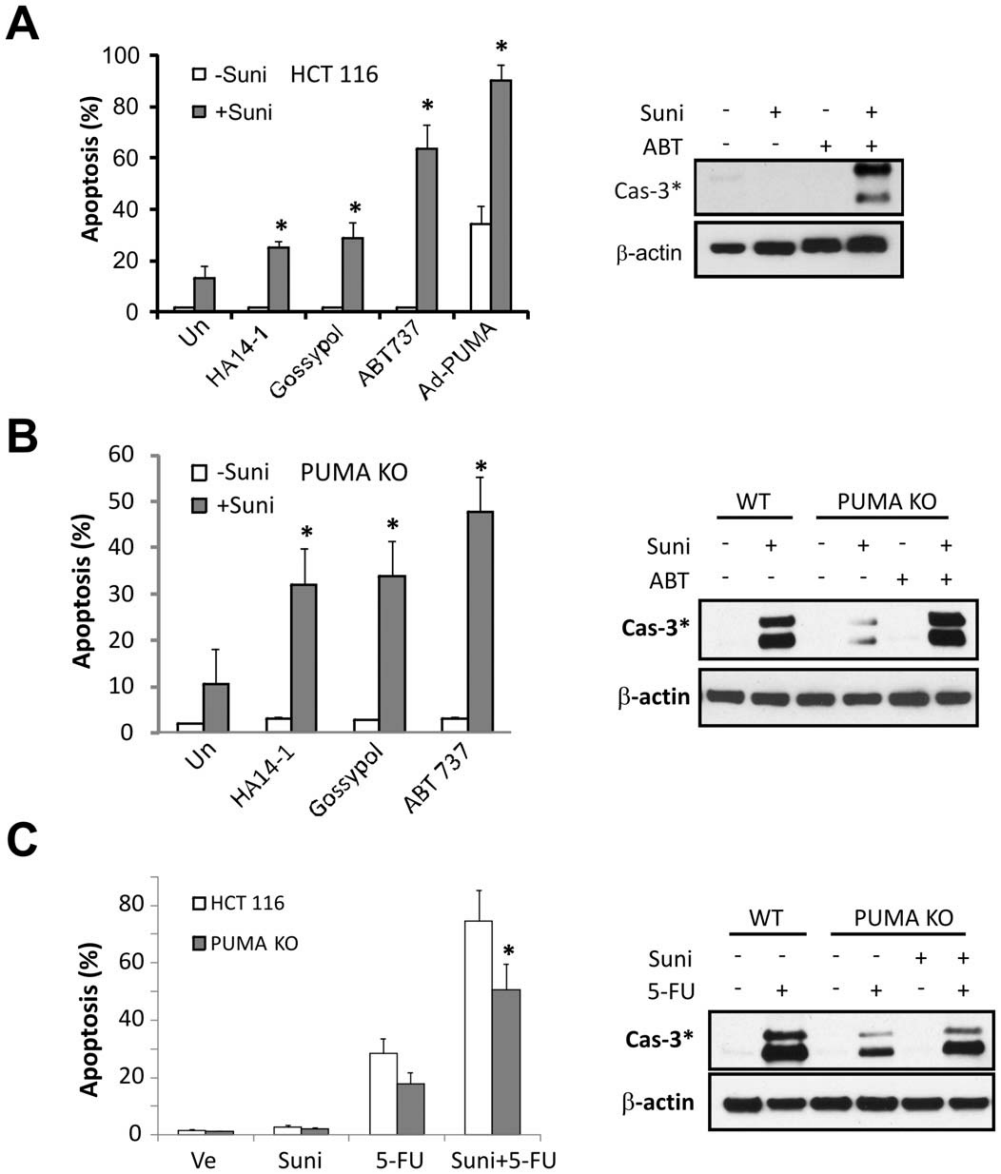
BH3 mimetics or elevated PUMA levels sensitize WT and *PUMA* KO cells to sunitinib-induced apoptosis. Apoptosis was determined by nuclear fragmentation assay and activation of caspases. All data on apoptosis were obtained from 3 independent experiments while representative western blots are shown. (**A**) HCT 116 cells were treated with 10 µM sunitinib, 5 µM HA14-1, 5 µM Gossypol, 1 µM ABT-737, 20 MOI Ad-PUMA (0.2 µl/mL) alone, or their combination for 48 hours. *, *P*<0.05, combination *vs.* single agent. *Right*, processed caspase-3 was detected by Western blotting. (**B**) HCT 116 *PUMA* KO cells were treated with 12 µM sunitinib, 5 µM HA14-1, 5 µM Gossypol, 1 µM ABT-737, 20 MOI Ad-PUMA (0.2 µl/mL) alone, or their combination for 48 hours. *, *P*<0.05, combination *vs.* single agent. *Right*, processed caspase-3 was detected by Western blotting. (**C**) HCT 116 WT and *PUMA* KO cells were treated with 10 µM sunitinib, 30 µg/ml 5-FU either alone or in combination for 48 hours. *, *P*<0.05, KO *vs.* WT. *Right*, processed caspase-3 was detected by Western Blotting.

### Statistical Analysis

Statistical analysis was carried out using GraphPad Prism IV software. All P-values were calculated by the student’s t-test, and P<0.05 was considered significant. Means ± one standard deviation (SD) were displayed in figures where applicable.

## Results

### PUMA is Induced by Sunitinib in Colon Cancer Cells

The expression of PUMA is low in most unstressed cells, and is induced by various genotoxic and non-genotoxic stresses [23]. To determine a potential role of PUMA in sunitinib-induced response, we analyzed the levels of PUMA, p21 and p53 before and after treatment in five colon cancer cell lines. Three of these lines, HCT 116, RKO and SW 48 contain WT *p53* while two lines HT 29 and SW 480 contain mutant *p53*. PUMA was induced by sunitinib in all five cell lines (Fig. 1A). PUMA induction was dose-dependent and occurred in both HCT 116 WT and *p53* knockout (KO) cells (Fig. 1B). Basal levels of PUMA were lower in *p53* knockout cells compared to WT cells as previously reported (Fig. 1B) [15]. p53 and p21 were also induced by sunitinib treatment. *PUMA* mRNA was rapidly induced by sunitinib, preceding protein induction (Fig. 1C and 1D). These data suggest that PUMA is transcriptionally induced by sunitinib independent of p53 in colon cancer cells.

**Figure 6:**
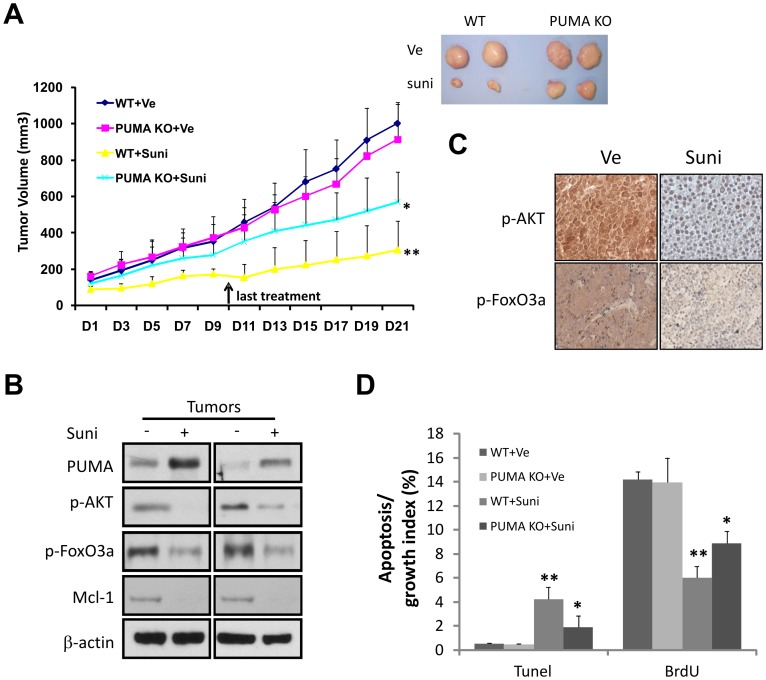
PUMA mediates therapeutic responses to sunitinib *in vivo*. (**A**) *Left*, growth curve of HCT 116 WT and *PUMA* KO tumors treated with sunitinib (Suni, oral gavage, 80 mg/kg/mouse, daily for 10 days) or vehicle (ve, citrate buffer pH4.7) for 21 days. Tumor volume was measured every other day. N = 7 per group. **, *P*<0.01, WT+Ve *vs.* WT+Suni: *, *P*<0.05, WT+Suni *vs. PUMA* KO+ Suni. *Righ*t, a representative picture of WT and *PUMA* KO tumors treated as indicated at day 21. (**B**) Sunitinib induced PUMA in xenograft tumors. The levels of indicated proteins in four randomly selected WT KO tumors were detected by Western blotting. (**C**) The levels of p-AKT and p-FoxO3a in WT tumors 24 hours following the third dose were analyzed by IHC. Representative pictures are shown. (**D**) *PUMA* deficiency inhibited sunitinib-induced apoptosis and growth suppression in xenograft tumors. Paraffin sections of the HCT 116 tumors 24 hours after the third injection were subjected to TUNEL and BrdU staining to quantitate apoptosis and proliferation, respectively. The index of TUNEL-positive or BrdU-labeled cells was calculated. **, *P*<0.01, WT+Ve *vs.* WT+Suni: *, *P*<0.05, WT+Suni *vs. PUMA* KO+ Suni.

### PUMA Mediates Sunitinib-induced Apoptosis

To examine a potential role of PUMA in sunitinib-induced apoptosis, we compared the responses of HCT 116 cells with isogenic *PUMA* knockout (KO) cells [15]. *PUMA* KO cells were highly resistant to sunibinib-induced apoptosis (Fig. 2A). As expected, activation of caspase-3 and -9, or cytosolic release of cytochrome c or Smac was impaired in *PUMA* KO cells, but not in *p53* KO cells (Fig. 2B). In long-term colony formation assays, *PUMA* KO cells generated over 4 times more colonies than WT cells (Fig. 2C). PUMA was also significantly induced by sunitinib in *p53* mutant DLD 1 cells while *PUMA* deficiency significantly blocked sunitinib-induced apoptosis and caspase activation in these cells (Fig. 2D). We also examined the levels of several BH3-only proteins and antiapoptotic Bcl-2 family members after sunitinib treatment. No consistent change was observed in most of them, except for a rapid Mcl-1 downregulation and a delayed induction of Bim after 24 hours, in HCT 116 cells (Fig. S1A). However, little or no Mcl-1 downregulation or Bim induction was observed in DLD1 cells (Fig. S1B). Interestingly, the levels of several Bcl-2 family members increased in *PUMA* KO cells compared to WT cells, perhaps reflecting their degradation by proteases activated during the treatment and apoptosis (Fig. S1B). These results suggest that PUMA plays a key role in the apoptotic responses to sunitinib in colon cancer cells.

### The Mechanism of PUMA Induction by Sunitinib

The PI3K/AKT pathway is a common effector downstream of multiple kinases targeted by sunitinib. We therefore examined AKT activation in a time course experiment following sunitinib treatment, and found AKT de-phosphorylation within minutes (Fig. 3A). FoxO3a is a transcription factor and well-established target of AKT, and its phosphorylation by AKT leads to inactivation and nuclear exclusion. Sunitinib treatment led to a rapid de-phosphorylation of FoxO3a, yet had no obvious effect on total FoxO3a levels (Fig. 3A). It is noted that changes in FoxO3a and AKT phosphorylation, or *PUMA* mRNA are dynamic and transient; while induction of PUMA protein is persistent and uniform across different cell lines (Figs. 1 and 3A). Exogenous expression of active AKT suppressed PUMA induction by sunitinib (Fig. 3B). The induction of PUMA was significantly inhibited by *FoxO3a* knockdown by either transient expression of siRNA or stable expression of shRNA in both WT and *p53* KO HCT 116 cells (Fig. 3C and 3D). *p53* KO cells were used to reduce p53-depedent PUMA induction by transfection.

We next determined whether FoxO3a directly activates *PUMA* transcription by chromatin Immunoprecipitation (ChIP) assay. Two FoxO3a binding sites are located in the first intron of *PUMA* [19]. The recruitment of FoxO3a to the *PUMA* promoter containing these sites increased as early as 8 hours following sunitinib treatment (Fig. 4A). Using reporter assays, we found that mutations in the FoxO3a binding sites significantly reduced PUMA reporter activity following sunitinib treatment (Fig. 4B). Furthermore, *FoxO3a* stable knockdown cells were found to be resistant to sunitinib-induced apoptosis (Fig. 4C). Mcl-1 levels were restored by *FoxO3a* siRNA in sunitinib-treated cells (Figs. 1D and 3C, S1), suggesting its degradation could be an additional mechanism of sunitinib-induced apoptosis in HCT 116 cells. Sunitinib-induced apoptosis occurred on other CRC lines including HT29, and was suppressed by overexpression of Mcl-1 or Bcl-2 (Figs. 4D and S2). These data indicate that FoxO3a regulates PUMA induction and the mitochondrial pathway in sunitinib-induced apoptosis.

### BH3 Mimetics or Elevated PUMA Levels Sensitize Colon Cancer Cells to Sunitinib

The above observations predict high levels of PUMA, BH3-only proteins, or small molecule BH3 mimetics sensitize cancer cells to sunitinib-induced apoptosis. Several BH3 mimetics including HA14-1, gossypol and ABT-737, and PUMA adenovirus (Ad-PUMA [15]), were able to sensitize HCT 116 cells to sunitinib. These agents alone induced little or limited apoptosis (Fig. 5A). Interestingly, BH3 mimetics induced significant apoptosis and caspase activation in *PUMA* KO cells when combined with sunitinib (Fig. 5A and 5B). DNA damaging agents such as 5-FU induces PUMA and Noxa in p53 WT cells [14], [32], [33], [34]. 5-FU also synergized with sunitinib to induce apoptosis in HCT 116 cells (Fig. 5C), which is associated with enhanced PUMA induction (Fig. S3). This synergy was attenuated but not blocked in *PUMA* KO cells (Fig. 5C) perhaps due to modulations of other Bcl-2 family members via both p53-dependent and independent mechanisms. These data demonstrate that elevated levels of PUMA or BH3 mimetics can enhance apoptotic responses to sunitinib, even in apoptosis-resistant cells.

### PUMA Mediates the Therapeutic Responses to Sunitinib in Xenograft Models

To assess whether PUMA modulates therapeutic responses *in vivo*, WT and *PUMA* KO HCT 116 cells were injected subcutaneously into the flanks of BALB/c (nu/nu) nude mice to establish xenografts. Compared to the vehicle, sunitinib treatment resulted in 62% and 38% growth inhibition in HCT 116 WT and *PUMA* KO tumors, respectively (Fig. 6A). Differences between *PUMA* genotypes were statistically significant in the sunitinib arm (P<0.01), but not in the vehicle arm regarding efficiency or growth rate in tumor establishment (Fig. 6A).

PUMA was significantly induced in WT tumors from sunitinib-treated mice (Fig. 6B). Modulations of Mcl-1, phosphorylated FoxO3a and AKT were also observed (Fig. 6B and 6C). Analyzing tumor sections from mice one day after the third sunitinib administration (day 4), we found a significantly lower rate of apoptosis and higher rate of cell proliferation in *PUMA* KO tumors when compared to WT tumors (Figs. 6D and S4A). Active caspase-3 staining confirmed significantly reduced apoptosis in *PUMA* KO tumors (∼80%), compared to WT tumors treated identically (Fig. S4B). These results demonstrate that the therapeutic response to sunitinib *in vivo* is mediated by PUMA-dependent apoptosis.

## Discussion

Emerging evidence suggests that induction of apoptosis is an important mechanism of a wide variety of anticancer agents [2], [10], [35]. Evasion of cell death is a hallmark of cancer and an important contributor to therapeutic resistance [2], [3]. In addition to well-documented effects of sunitinib in inhibiting tumor angiogenesis [6], [7], [36], [37], our work demonstrates that sunitinib exhibits a strong pro-apoptotic activity in colon cancer cells via PUMA induction through transcription factor FoxO3a, but not p53, NF-κB p65 or p53 homologue p73 and p63. Sunitinib-induced apoptosis is associated with the induction of Bim or down regulation of Mcl-l in some colon cancer cell lines we tested. Earlier work demonstrated the involvement of Bim and STAT3 during sunitinib-induced apoptosis in other cells types [38], [39], [40]. Together, these data suggest that induction of BH3-only proteins might be a common mechanism underlying sunitinib-induced cancer cell killing that might be affected by status of various kinases, and different BH3-only proteins might be important in different cells types.

A number of more selective VEGFR inhibitors were also found to induce PUMA and apoptosis in colon cancer cells (data not shown), supporting a non-angiogenic role of anti-VEGFR therapies. It will be important to determine whether sunitinib-induced apoptosis is mediated by PUMA or other BH3-only proteins in other solid tumors such as renal cancer and GISTs, and their potential role in the apoptotic responses to other VEGFR and PDGFR inhibitors. Reduced sensitivity to sunitinib was suggested to be linked to mutations in *KIT* or *VEGFR/FDGFR* or in other RTKs, as well as decreased expression of soluble VEGF receptors (sVEGFs) [6], [7], which can suppress cell death or promote survival [2], [5]. In addition, inhibition of tumor angiogenesis or other components in the microenvironment might indirectly activate cell death pathways [2]. The use of isogenic cell lines as we did here could be particularly useful in understanding drug targets and mechanisms [41].

Despite the excitement in the development of agents targeting oncogenic kinases, clinical data demonstrate that most of these agents are generally efficacious only in a minor fraction of patients [4], [5]. One major change is to identify biomarkers to help patient selection and stratification. Our data showed that PUMA persists 24–48 hours after sunitinib treatment. In contrast, inhibition of AKT/FoxO3a is more transient and recovers in hours, likely reflecting secondary and survival attempts in tumor cells following activation of apoptotic signaling. These findings potentially explain why upstream signaling molecules are less suitable as biomarkers, and suggest modulation of the mitochondrial death pathway might be a more useful readout for the overall therapeutic activity of anticancer agents. It may be particularly relevant to explore the regulation of Bcl-2 family members as they are rarely found mutated in solid tumors [2], [3], and can be functionally achieved by “BH3 profiling” as demonstrated recently in leukemia patients following chemotherapy [42], [43]. Current biomarkers of sunitinib include plasma levels of VEGF, (soluble) sVEGFR2, sVEGFR3, and sKIT [7], [8]. Interrogation of changes in PUMA and other Bcl-2 family members using biopsies collected before and after treatment would certainly be possible as well as informative. Therefore, a combination of markers monitoring tumor microenvironment, angiogenesis (non cell-autonomous), cell survival and death (cell-autonomous) might ultimately prove more useful.

Current treatment modalities provide limited benefits to patients with advanced colorectal cancer [1], and targeted agents might bring new hopes. A better understanding of their mechanisms of action and identification of biomarkers are expected to help guide their further development, clinical testing and use. Our work suggests that PUMA induction predicts the apoptotic responses to sunitinib in colon cancer cells, and provides potential strategies for combination therapies. In preclinical models, small molecule BH3 mimetics synergize with a wide variety of anticancer agents, and generally have none-overlapping side effects with chemo-drugs or kinase inhibitors [7], [44], [45]. Therefore, it is possible that carefully designed combinations will provide more effective treatment and long-term management of cancer [2].

In summary, our study provides a novel anti-tumor mechanism of sunitinib. In line with recent findings on several BH3-only proteins in targeted therapies [13], [19], [20], [46], [47], [48], we propose that induction, rather than the steady-state levels, of selective BH3-only proteins as potential biomarkers in both treatment naive and none-naïve patients. This is an important concept as many kinases inhibitors are used in heavily pretreated patients whose cancer might already have elevated levels of BH3-only proteins. It will be important to determine whether BH3 mimetics or certain chemo-drugs can improve the efficacies of kinase inhibitors in preclinical models and clinical trials.

## Supporting Information

Figure S1 **The expression of Bcl-2 family of proteins in colon cancer cells following sunitinib treatment.** (**A**) HCT 116 cells were treated with 15 µM sunitinib for the indicated times. The levels of indicated BH3-only and anitapoptotic Bcl-2 members were analyzed by Western blotting. β-actin was used as a control for loading in Western blotting. (**B**) WT or *PUMA* KO DLD1cells were treated with 30 µM sunitinib for 48 hours. The levels of indicated Bcl-2 family members were analyzed by Western blotting. β-actin was used as control for loading in Western blotting. Bcl-2 was not detected in these cells.
(JPG)

Figure S2 **Expression of Mcl-1 or Bcl-2 suppresses sunitinib-induced apoptosis.** (**A**) HT 29 cells were transfected with a Mcl-1 expression construct or empty vector followed by 15 µM sunitinib treatment for 48 hours. Apoptosis was analyzed by nuclear fragmentation assay. (**B**) The expression of Mcl-1 (Flag-tagged) was confirmed by Western blotting. β-actin was used as control for loading. Un: untreated. (**C**) HCT 116 and HT 29 cells were transfected with a Bcl-2 expression construct or empty vector followed by 15 µM sunitinib treatment for 48 hours. Apoptosis was analyzed by nuclear fragmentation assay. *, *P*<0.05, transfection of Mcl-1 or Bcl-2 *vs.* vector.
(JPG)

Figure S3 **5-FU and sunitinib synergized to induce PUMA expression.** HCT 116 cells were treated with 10 µM sunitinib, 30 µg/ml 5-FU alone, or in combination for 24 hours. The expression of PUMA was analyzed by Western Blotting.
(JPG)

Figure S4 ***PUMA*** **deficiency impaired sunitinib-induced apoptosis and growth suppression** ***in vivo*** **.** (**A**) Paraffin sections of the HCT 116 tumors with indicated genotypes 24 hours following the third injection were analyzed by H&E staining, TUNEL staining (*red*) for apoptosis, and BrdU incorporation (*red*) for proliferation. The nuclei were counterstained DAPI (*blue*). Magnification, ×400. (**B**) Apoptosis was determined by active caspase-3 staining in the tumors with indicated treatments as in (A). Four high power 400x fields were used for each determination. **, *P*<0.01, WT+Suni *vs. PUMA* KO+ Suni.
(JPG)
