# Supplementary figures and images for: The Multi-Targeted Kinase Inhibitor Sunitinib Induces Apoptosis in Colon Cancer Cells via PUMA
Source: PLoS One. 2012 Aug 17;7(8):e43158. doi: 10.1371/journal.pone.0043158 (PMC3422222; doi:10.1371/journal.pone.0043158)

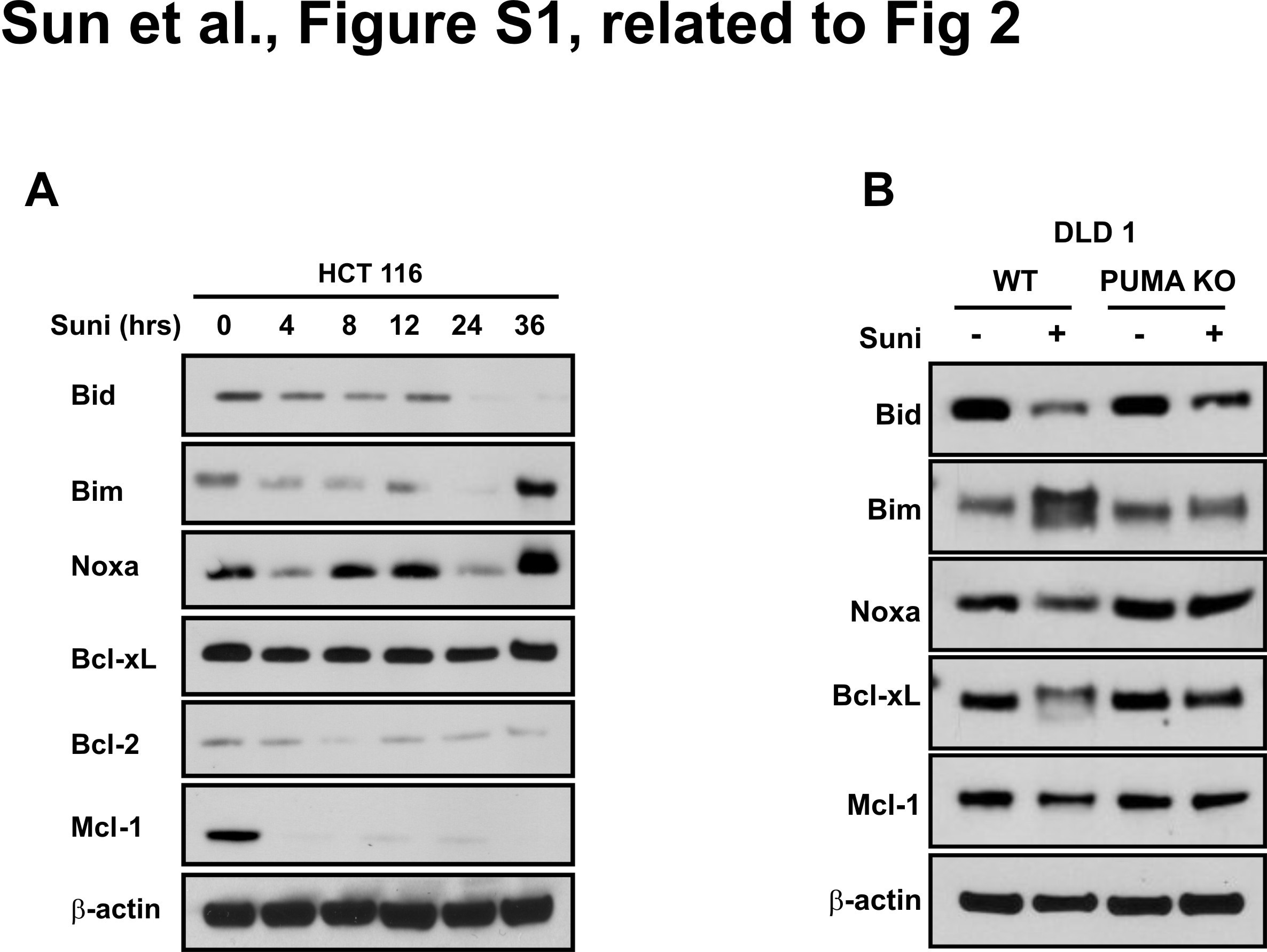

Supplement: Figure S1 — The expression of Bcl-2 family of proteins in colon cancer cells following sunitinib treatment. (A) HCT 116 cells were treated with 15 µM sunitinib for the indicated times. The levels of indicated BH3-only and anitapoptotic Bcl-2 members were analyzed by Western blotting. β-actin was used as a control for loading in Western blotting. (B) WT or PUMA KO DLD1cells were treated with 30 µM sunitinib for 48 hours. The levels of indicated Bcl-2 family members were analyzed by Western blotting. β-actin was used as control for loading in Western blotting. Bcl-2 was not detected in these cells. (JPG) [file pone.0043158.s001.jpg]

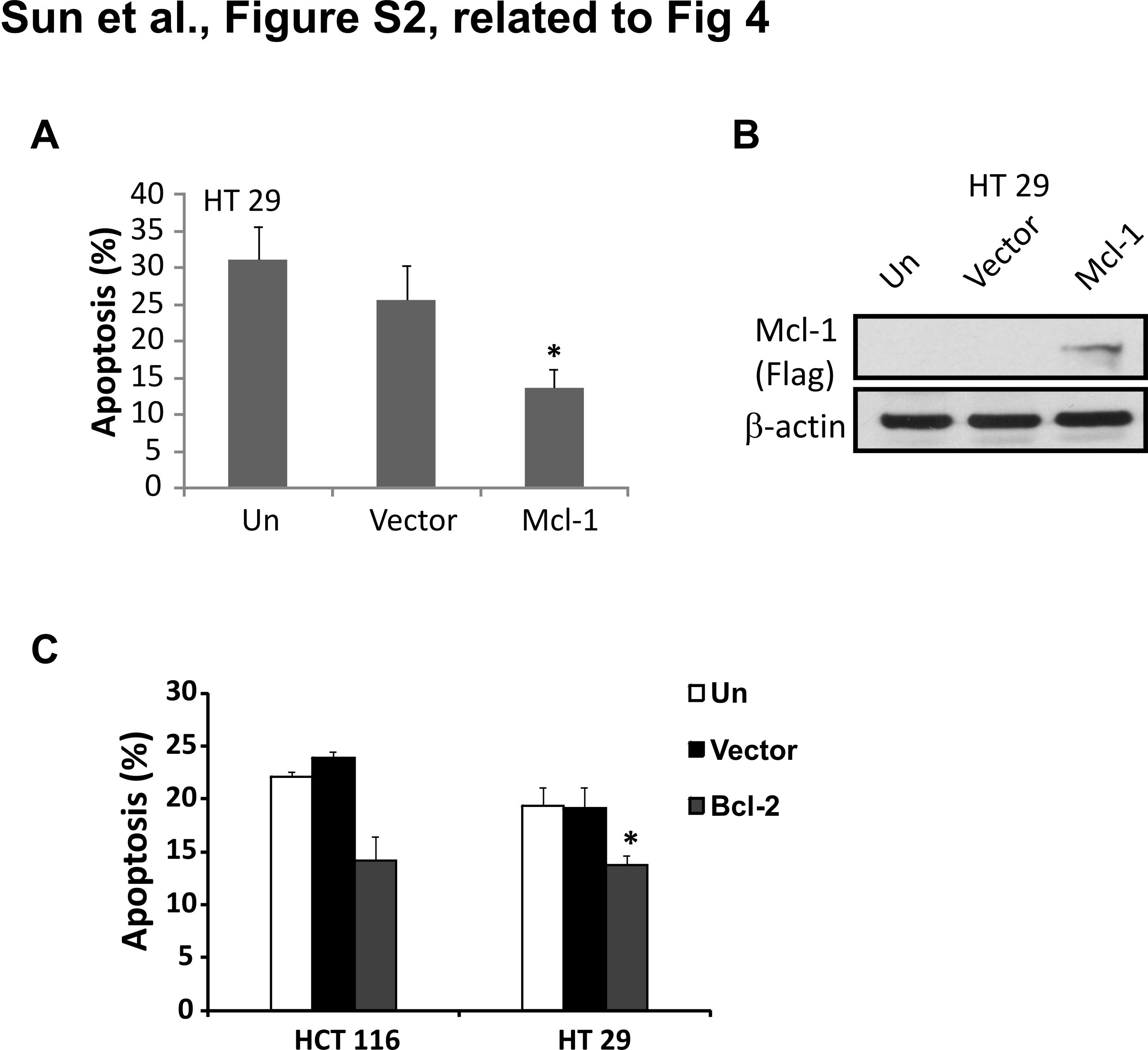

Supplement: Figure S2 — Expression of Mcl-1 or Bcl-2 suppresses sunitinib-induced apoptosis. (A) HT 29 cells were transfected with a Mcl-1 expression construct or empty vector followed by 15 µM sunitinib treatment for 48 hours. Apoptosis was analyzed by nuclear fragmentation assay. (B) The expression of Mcl-1 (Flag-tagged) was confirmed by Western blotting. β-actin was used as control for loading. Un: untreated. (C) HCT 116 and HT 29 cells were transfected with a Bcl-2 expression construct or empty vector followed by 15 µM sunitinib treatment for 48 hours. Apoptosis was analyzed by nuclear fragmentation assay. *, P<0.05, transfection of Mcl-1 or Bcl-2 vs. vector. (JPG) [file pone.0043158.s002.jpg]

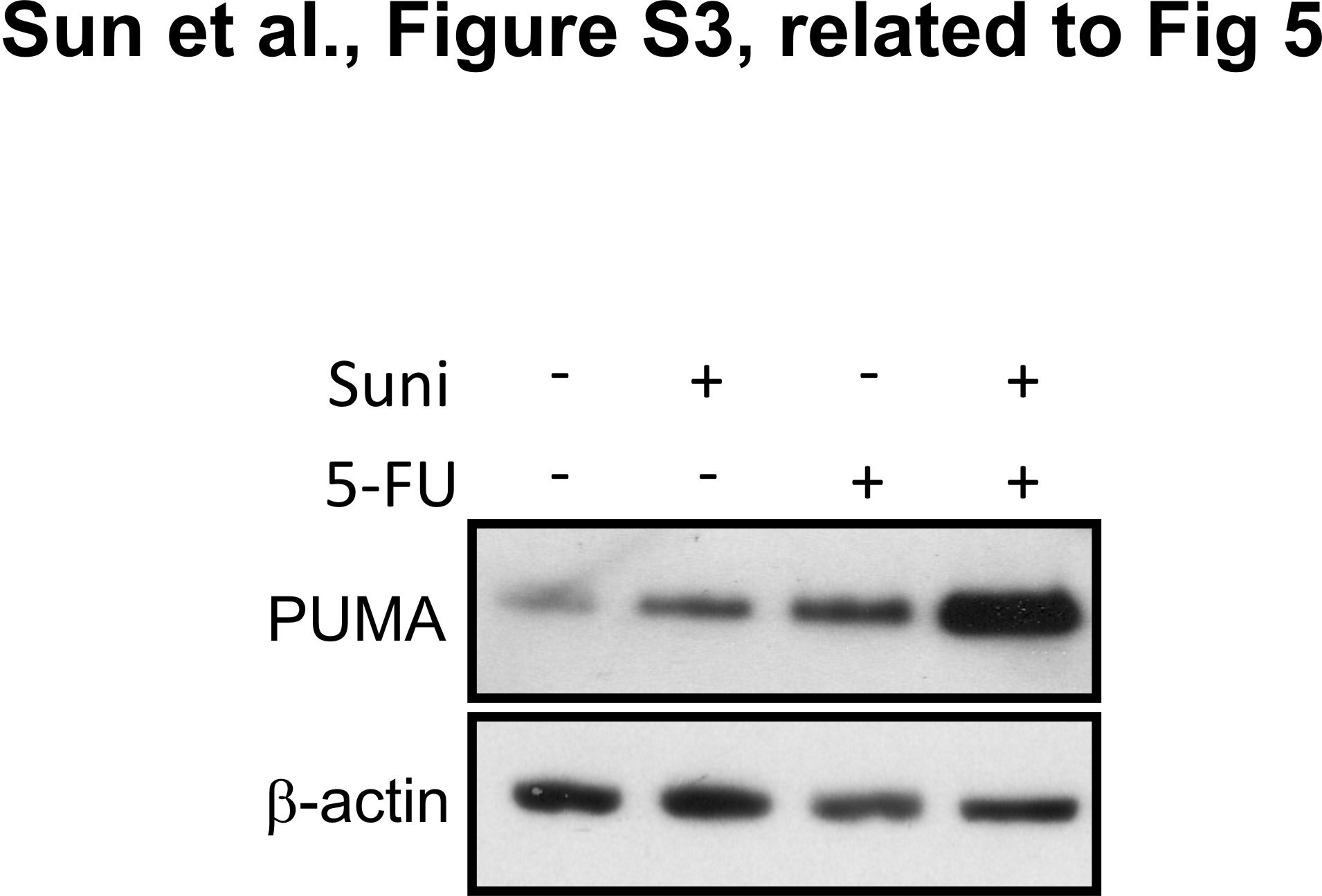

Supplement: Figure S3 — 5-FU and sunitinib synergized to induce PUMA expression. HCT 116 cells were treated with 10 µM sunitinib, 30 µg/ml 5-FU alone, or in combination for 24 hours. The expression of PUMA was analyzed by Western Blotting. (JPG) [file pone.0043158.s003.jpg]

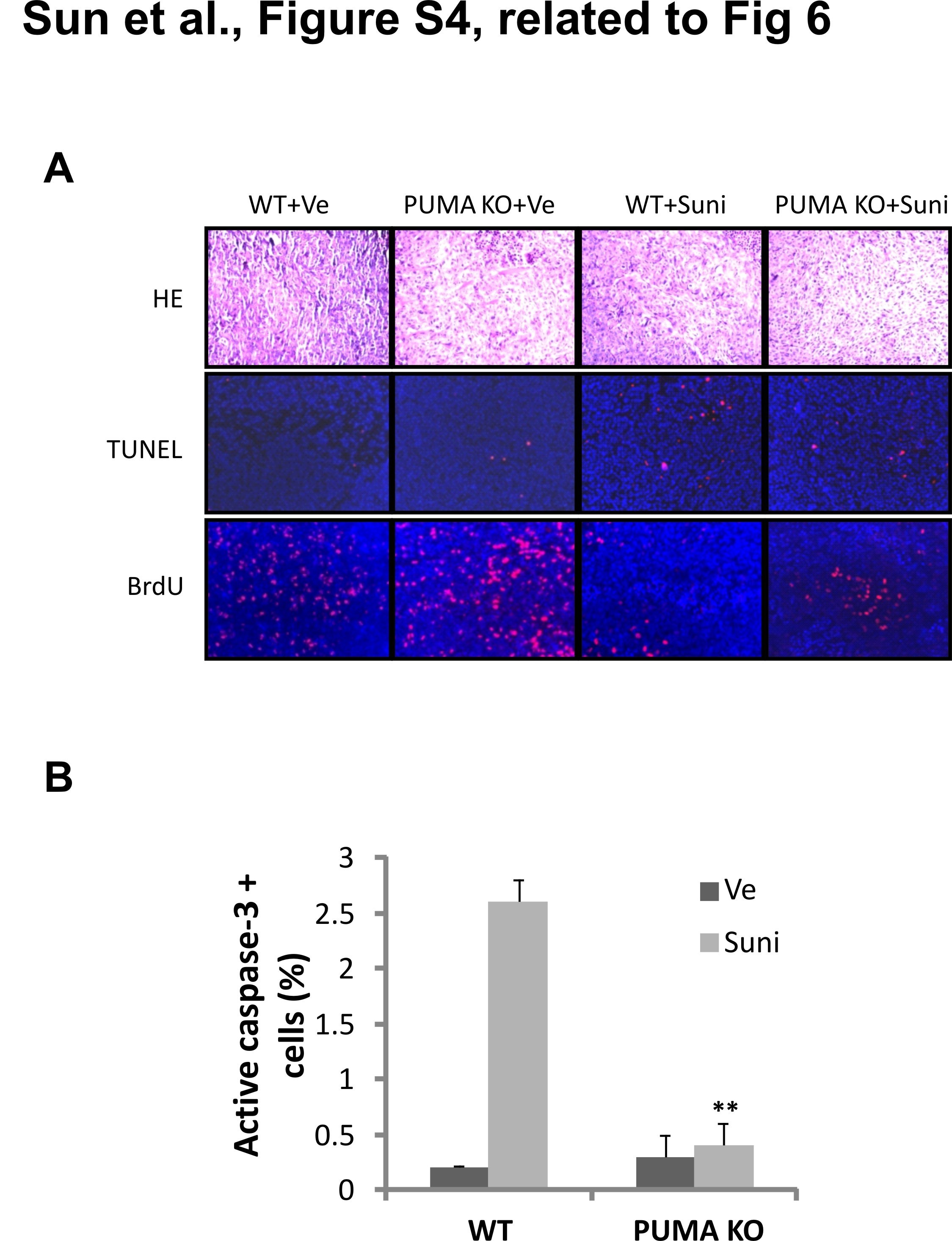

Supplement: Figure S4 — PUMA deficiency impaired sunitinib-induced apoptosis and growth suppression in vivo . (A) Paraffin sections of the HCT 116 tumors with indicated genotypes 24 hours following the third injection were analyzed by H&E staining, TUNEL staining (red) for apoptosis, and BrdU incorporation (red) for proliferation. The nuclei were counterstained DAPI (blue). Magnification, ×400. (B) Apoptosis was determined by active caspase-3 staining in the tumors with indicated treatments as in (A). Four high power 400x fields were used for each determination. **, P<0.01, WT+Suni vs. PUMA KO+ Suni. (JPG) [file pone.0043158.s004.jpg]
